# Effect of Annealing Process on the Microstructure and Texture of Cold-Rolled High-Purity Al-0.5%Cu Plates

**DOI:** 10.3390/ma15103489

**Published:** 2022-05-12

**Authors:** Kuiwen Yuan, Jiaxin Chen, Dan Yang, Zhiqing Zhang

**Affiliations:** College of Materials Science and Engineering, Chongqing University, Chongqing 400044, China; yuankw1229@163.com (K.Y.); chenjiaxin9809@163.com (J.C.); yangdan9851@163.com (D.Y.)

**Keywords:** Al-0.5%Cu alloy, annealing process, recrystallization, microstructure, texture

## Abstract

As a kind of typical high stacking fault energy materials, recrystallization behavior of high purity Al-0.5%Cu alloy is significantly influenced by the annealing process. In this study, different heating rate, target temperature, and holding time were discovered to have profound impact on the microstructures and textures of Al-0.5%Cu plates. Electron backscatter diffraction (EBSD), scanning electron microscope (SEM), and X-ray diffraction (XRD) were utilized for analyzing the evolution of the microstructure and texture in the subsequent microstructural characterization. Vickers hardness tests were employed for measuring hardness of specimens. The results showed that no obvious recrystallization was observed at lower temperature and the composition of texture influenced by rising temperature, heating rate affected initial recrystallization temperature, grain size, and strength of textures. After recrystallizing completely, the size of microstructures and the distribution of textures had little change with the extension of holding time.

## 1. Introduction

Al-Cu alloy was widely used in automobile industry, aerospace field and integrated circuit field [1,2,3]. In recent years, with the development of IC industry, the demand of Al-Cu alloy is increasing, which is manufacturing material as sputtering target materials [4,5,6]. At present, different annealing processes are adopted to improve the microstructure of Al-Cu alloy and optimize the properties of the sputtering target material. The purity, grain size and texture of Al-Cu alloy have great influence on sputtering target material, and the materials used in previous studies were commercial level alloy which means that these materials had high trace element compositions [7,8,9]. As we all know, even low-level trace element pin dislocations migration and consequently affect recrystallization behavior. In order to exclude influence of trace element on recrystallization behavior, high level purity Al-Cu alloy was used in this study.

High purity Al-0.5% Cu plate is a kind of aluminum alloy with high strength and corrosion cracking resistance [10]. AS a deformed material, this material is thermodynamically unstable because of its free energy is raised by the presence of dislocations and interfaces which are introduced during plastic deformation. Annealing is mainly to reduce the dislocation density caused by cold work hardening and improve the properties of alloy and refine the grain size [11,12,13,14]. Dislocations were diminished by recovery and recrystallization during annealing. It was believed that the stored energy of non-recrystallization materials is steadily reduced by recovery process [15,16], which retards recrystallization. The extent of this retardation depends on stacking fault energy of the material, amount of deformation, annealing temperature, and heating rate.

There have been several studies investigating the annealing process of aluminum alloy. Fang, H. [17] found that the Al-Mg-Sc-Zr alloy was able to precipitate as a nanoscopic second phase Al_3_ (Sc, Zr), which has the effect of hindering grain boundary movement and strongly inhibiting the recrystallization of the alloy. Shuai, L. F. et al. [18] described the evolution of the recrystallization texture of Al-0.3%Cu alloy annealed at different temperatures for one hour. Engler, O. [19] had studied that the microstructure and texture evolution of the sample with coarse initial grain size during annealing was obviously different from that of the samples with fine initial grain size. Shen, F. et al. [20] investigated the texture and microstructure evolution of the 2524 Al-Cu-Mg cold-rolled sheet annealed at 140–560 °C for one hour. They reported that different annealing progresses had significant influence on the microstructure and texture evolution of aluminum alloy.

Compared with the works which researchers had done on effects of deformation reduction and annealing type on recrystallization behavior of high stacking fault energy materials [21], less attention was put on the effect of heating rate, target temperature and holding time on recrystallization behavior. Therefore, the investigation of different annealing processes was of great significance to further understand the microstructures and properties of the alloy after annealing.

## 2. Materials and Methods

**Figure 1:**
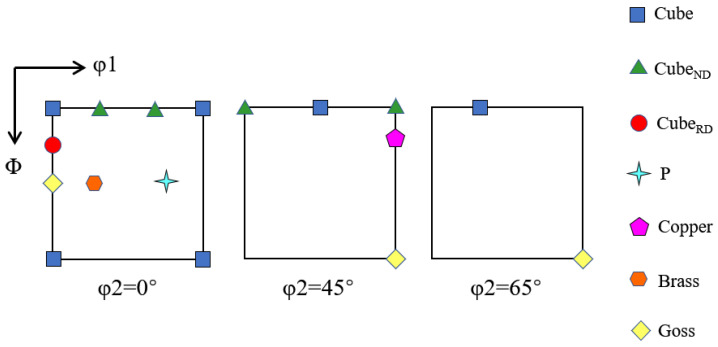
Typical texture (φ_2_ = 0°, 45°, 65°) position in FCC metals.

**Figure 2:**
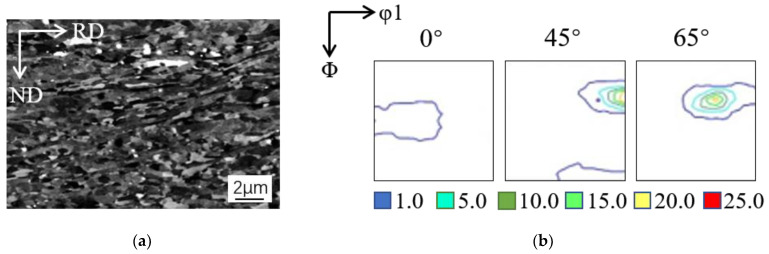
(**a**) SEM micrograph and (**b**) texture distribution (φ_2_ = 0°, 45°, 65°) of cold-rolled Al-0.5%Cu.

In the present investigation, a kind of high purity Al-0.5% Cu plate which had been forged to 20 mm in thickness followed by annealing was used as the start material. The aforementioned plate was cold rolled to 90% thickness reduction, and 20 mm × 40 mm strips were cut with the longer dimension parallel to rolling direction (RD). Three incomplete pole figures {111}, {200}, {220} were recorded, and the orientation distribution function (ODF) was calculated using series expansion method on Labotex. The typical texture position (φ_2_ = 0°, 45°, 65°) in ODFs and main texture components of FCC metals were shown in Figure 1 and Table 1, respectively. The cold-rolled sheets possessed typical deformed structure and strong β fiber rolling texture as shown in Figure 2a,b.

The continuous heat treatments were carried out using air furnace and oil bath, which were two most often used methods in material heat treatment [22,23,24]. Because air and oil have different thermal conductivities, the heating rate of air furnace and oil bath are different. Oil bath heating usually has higher heating rate than air furnace. In order to study the effect of annealing processed on recrystallization behavior, the cold-rolled sheets were divided into two groups of samples. A group of annealed samples were heated to the target temperatures in between 200–300 °C by air furnace at a heating rate of 20 °C/min (SH) and held for range from 2.5–80 min. The other group was annealed by oil bath at a higher heating rate of 200 °C/min (RH) to the same temperature range and holding time as the specimens mentioned above. All samples were immediately water-quenched after target temperatures and holding time of each heat treatment was reached. 

The electron backscatter diffraction (EBSD) technique was carried out in a TESCAN MIRA3 field emission scanning electron microscope, which was used to characterize the local microstructure of strips in transverse direction (TD). The microstructure of the alloy sheets in different conditions was characterized by scanning electron microscope (SEM), the polished samples were etched by Keller reagent. X-ray diffraction (XRD) technique was employed to investigate the texture evolution. The (1 1 1), (2 0 0) and (2 2 0) pole figures were measured up to maximum tilt angle of 70°. The ODFs were presented as plots of constant φ_2_ sections with iso-intensity contours in Euler space defined by Euler angles φ_1_, Φ and φ_2_. Vickers hardness of the as-rolled and annealed sheets were investigated at room temperature using a MH-5L model hardness tester with load of 200 g and dwell time of 10 s, 15 measurements in center region of specimens were observed for each condition to get more accurate evaluation.

## 3. Results

### 3.1. Microstructure Evolution

**Figure 3:**
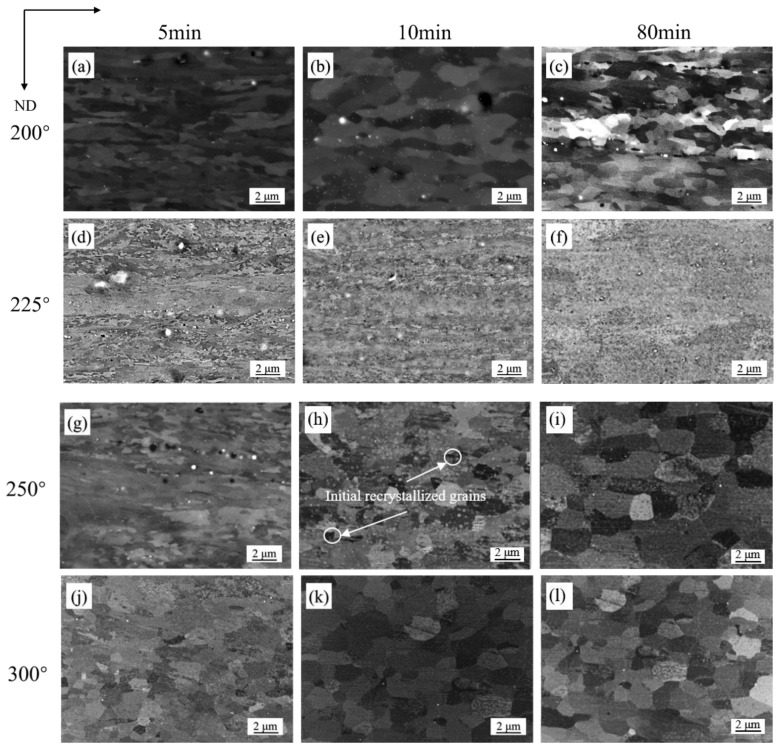
Microstructures of cold-rolled Al-0.5%Cu after annealing in SH: (**a**) 200 °C, 5 min; (**b**) 200 °C, 10 min; (**c**) 200 °C, 80 min; (**d**) 225 °C, 5 min; (**e**) 225 °C, 10 min; (**f**) 225 °C, 80 min; (**g**) 250 °C, 5 min; (**h**) 250 °C, 10 min; (**i**) 250 °C, 80 min; (**j**) 300 °C, 5 min; (**k**) 300 °C, 10 min; (**l**) 300 °C, 80 min.

Figure 3 shows the microstructure of cold-rolled sheets annealed at different temperatures for different holding time in SH. At 200 °C for 5 min, the micromorphology of the sample in SH (Figure 3a) was similar to that of the cold deformed microstructure (Figure 2a), which illustrated that this temperature was below or close to the initial recrystallization temperature. However, some uniform areas appeared with time increasing, that means recovery played important role in this stage (Figure 3b,c). With temperature and dwell time increasing, samples under lower heating rate had not recrystallized until 250 °C, the recrystallized grains could be observed clearly in Figure 3h. Once the recrystallized grains appear, they grew with increasing temperature and holding time (Figure 3h–l). Above 250 °C and 1 h, cold-rolled sheets were recrystallized fully with coarse grains (Figure 3i,l).

**Figure 4:**
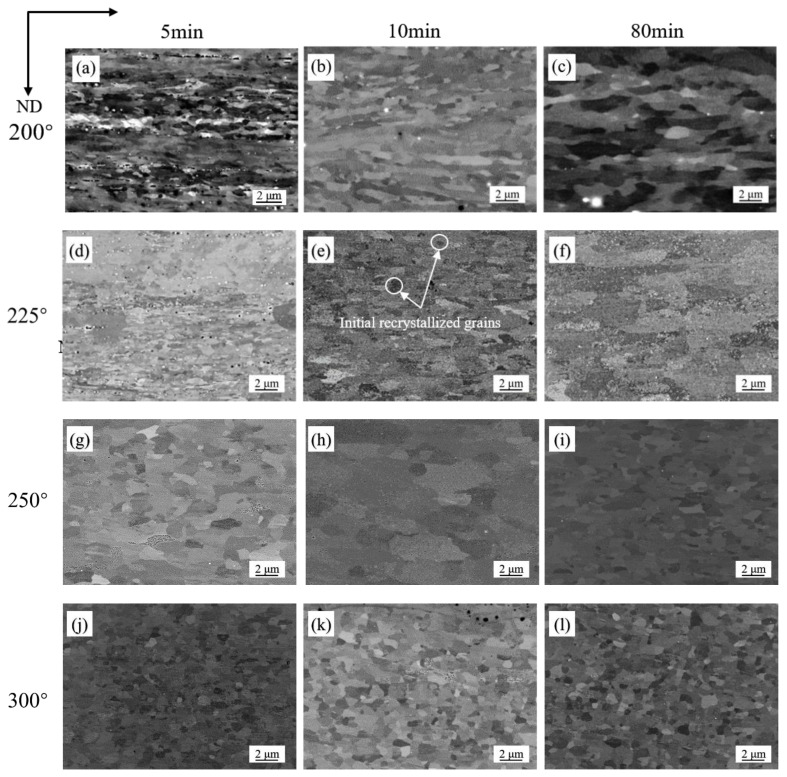
Microstructures of cold-rolled Al-0.5%Cu after annealing in RH: (**a**) 200 °C, 5 min; (**b**) 200 °C, 10 min; (**c**) 200 °C, 80 min; (**d**) 225 °C, 5 min; (**e**) 225 °C, 10 min; (**f**) 225 °C, 80 min; (**g**) 250 °C, 5 min; (**h**) 250 °C, 10 min; (**i**) 250 °C, 80 min; (**j**) 300 °C, 5 min; (**k**) 300 °C, 10 min; (**l**) 300 °C, 80 min.

**Figure 5:**
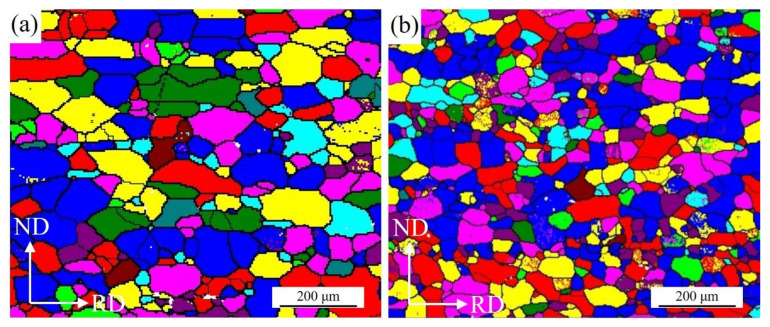
The EBSD map of the Al-0.5%Cu alloy sheets annealed at 300 °C for 80 min in two different heating rates: (**a**) SH; (**b**) RH.

**Figure 6:**
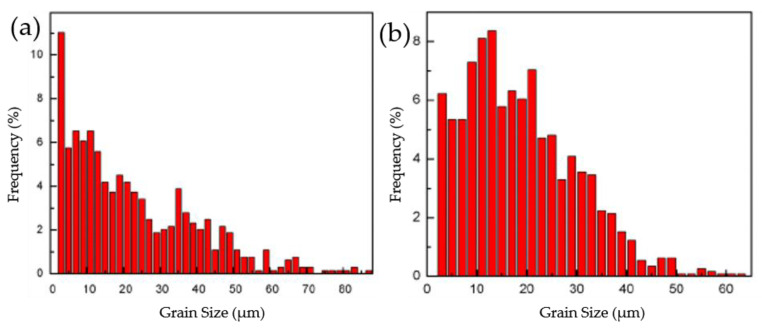
The grain size distribution of Al-0.5%Cu alloy sheets annealed at 300 °C for 80 min in two different heating rates: (**a**) SH; (**b**) RH.

The microstructures of cold-rolled sheets annealed at different temperatures for 5, 10 and 80 min in RH were presented in Figure 4. It can be seen from the figures that, different from samples heated slowly, the samples heated rapidly recrystallized at lower temperature. At 200 °C for 10 min, the cold-rolled plate had started to recrystallize, leading to a few newly recrystallized grains (Figure 4b). At 200 °C for 80 min, a number of recrystallized grains were observed and the amount of rolled texture was small at this time (Figure 4c). At 250 °C for 80 min, the sample was fully recrystallized with regular grain structures (Figure 4i). After completing recrystallization, the size of recrystallized grains increased slightly with annealing time (Figure 4j–l). Temperature, holding time and heating rate all affected recrystallization behavior, higher temperature accelerated recrystallization and longer holding time promoted growth of recrystallization nuclei. Figure 5 shows the EBSD maps of samples under two different heating rates at 300 °C for 8 min, it can be seen clearly that the grain size of RH specimen was finer. Figure 6 shows the grain size distribution of the samples in SH and RH treatment at 300 °C for 8 min. The average grain size of the sample in RH is 15.9 μm and the square deviation of grain distribution is 11.6, while that of the SH sample is 23.7 μm and 17.5. The main difference in between SH and RH was the size of the recrystallized grains. In the case of SH, coarse grains were exhibited in the specimens while finer equiaxed recrystallized grains were obtained in RH.

### 3.2. Hardness Properties

**Figure 7:**
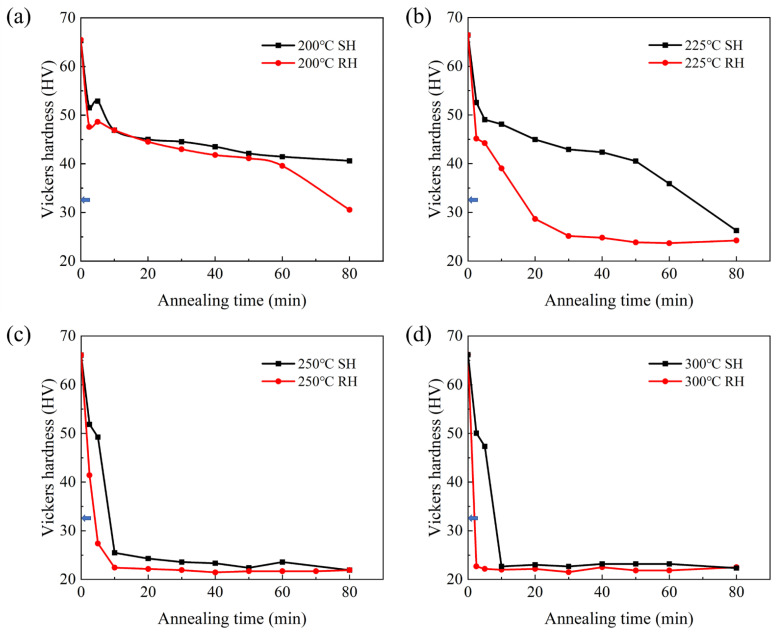
Hardness curves of cold-rolled Al-0.5%Cu annealed in different heating rate and heated to different target temperatures for different holding time, the arrows point to the original hardness: (**a**) 200 °C; (**b**) 225 °C; (**c**) 250 °C; (**d**) 300 °C.

Figure 7 shows typical hardness curves of cold-rolled Al-0.5%Cu annealed in SH and RH to different temperatures for different holding time. As can be seen from Figure 6, the original hardness of the sample was 33 HV, but after cold rolling deformation, the hardness increased to 66 HV. The hardness of all cold-rolled samples decreased after annealing treatment, and the hardness curves of samples annealed at 200 °C, 225 °C and 250 °C in SH and RH were obviously different. Two groups of curves in Figure 7a exhibited the hardness of RH samples and SH samples decreased at a similar rate in the first 2.5 min, which revealed that recovery proceeded at this stage in both of two conditions. Additionally, the hardness of SH samples decreased slowly in the subsequent time, while the hardness of RH samples decreased significantly after heating for 60 min. At 225 °C, the hardness curves of samples with RH and SH treatment were obviously different. The hardness of sample in RH treatment dropped from 66 to 28 HV in 20 min, and there was only little change after heating for more than 30 min, which indicated that RH samples had nearly completed recrystallization. However, the hardness of SH samples had not changed clearly until 50 min. From the curves in Figure 7c, hardness of samples in two different heating rates was close to equal and stable after 10minutes and hardness had little change with time increasing, which could be attributed to recrystallized completely. The similar tendency was found in hardness curves of 300 °C annealed samples, and hardness of samples dropped faster than that of 250 °C annealed one, which meant higher temperature could promote recrystallization. After recrystallization completed, hardness of all samples had little change with the increasement of time.

As a high stacking fault energy material [25,26], recovery of Al alloy is strong during annealing. At lower temperature, effect of recovery was obvious, but the stage of recovery decreased with temperature increasing. When the cold-rolled sheets started to recrystallize, hardness decreased sharply [27]. The recrystallization temperature of cold-rolled Al-0.5%Cu was affected by heating rate and temperature. Based on the hardness curves and observation of microstructures, it was determined that in the case of SH, the recrystallization started at about 250 °C, while recrystallization started at about 200 °C in the other case.

### 3.3. Texture Evolution

**Figure 8:**
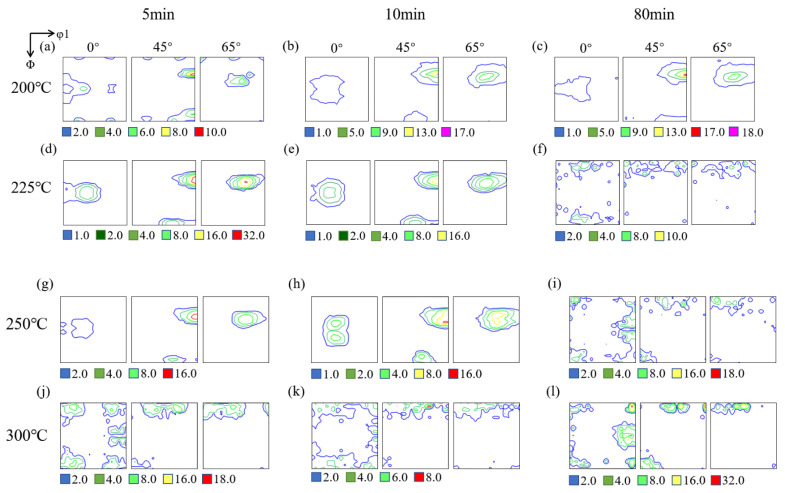
Textures evolution of cold-rolled Al-0.5%Cu samples after annealing in SH, the different colored boxes below represent the intensity of textures: (**a**) 200 °C, 5 min; (**b**) 200 °C, 10 min; (**c**) 200 °C, 80 min; (**d**) 225 °C, 5 min; (**e**) 225 °C, 10 min; (**f**) 225 °C, 80 min; (**g**) 250 °C, 5 min; (**h**) 250 °C, 10 min; (**i**) 250 °C, 80 min; (**j**) 300 °C, 5 min; (**k**) 300 °C, 10 min; (**l**) 300 °C, 80 min.

**Figure 9:**
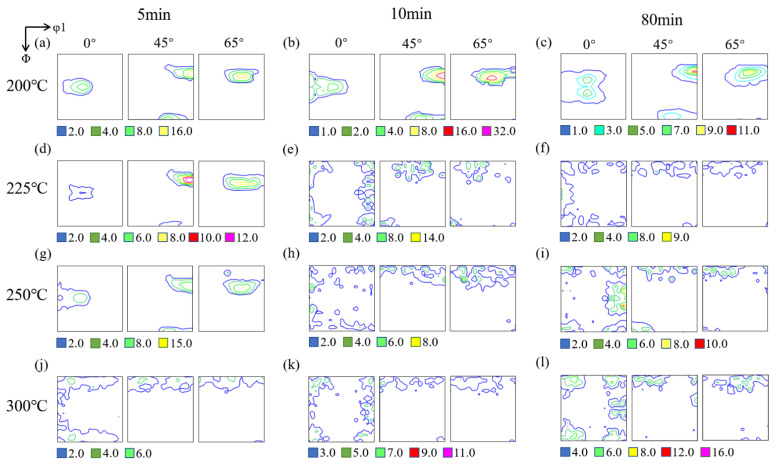
Textures evolution of cold-rolled Al-0.5%Cu samples after annealing in RH, the different colored boxes below represent the intensity of textures: (**a**) 200 °C, 5 min; (**b**) 200 °C, 10 min; (**c**) 200 °C, 80 min; (**d**) 225 °C, 5 min; (**e**) 225 °C, 10 min; (**f**) 225 °C, 80 min; (**g**) 250 °C, 5 min; (**h**) 250 °C, 10 min; (**i**) 250 °C, 80 min; (**j**) 300 °C, 5 min; (**k**) 300 °C, 10 min; (**l**) 300 °C, 80 min.

Figure 8 and Figure 9 illustrated the ODF sections (φ_2_ = 0°, 45°, 65°) of the crystallographic texture for the cold-rolled sheets annealed in different processes. After annealing at 200 °C for 5 min, intensity of the textures in both heating rates decreased, but the intensity of RH samples had a faster rate. The maximum texture intensity of RH samples decreased from 28 to 14, which indicated that the content of deformation texture decreased obviously; however, the type of textures did not change. It was evident that the texture was mainly consisted of rolling texture and recovery of samples did not alter the type of textures. With temperature increasing, the Goss and Brass texture almost disappeared. At 250 °C for 5 min, the type of texture under two different heating rate was still consistent with deformation texture. With the extension of holding time, recrystallization texture finally appeared, but the difference was that the texture type changed after 10 min of RH, while the type of texture changed after 80 min in SH. At 300 °C for 5 min, the texture types of both the RH samples and SH samples had changed, which illustrated that recrystallization texture had been formed in the materials under two heating rates. With the time increasing, after completing recrystallization at 300 °C for 80 min, the recrystallization textures were mainly characterized by Cube, Cube-_ND_ and P components, and the content of recrystallization texture was different in RH and SH specimens with the holding time increasing. The content of P texture in SH samples was higher than RH one, while more random texture generated in RH samples.

It can be seen from Figure 8 and Figure 9 that the intensity of deformation texture decreased gradually when the annealing temperature rises from 200–300 °C. The texture type of the samples in SH treatment did not change obviously at 225 °C, but the recrystallization texture content increased sharply at 250 °C and 300 °C, while the type of texture in RH changed significantly at 225 °C, 250 °C and 300 °C. The volume fractions of the Cube, Cube-_ND_, Goss, Brass and P components in fully recrystallized samples (300 °C, 80 min) in SH were 18.55, 6.26, 10.73, 4.40 and 11.49%, respectively, while they were 7.95, 5.59, 2.04, 2.32 and 3.35% in RH. At different temperatures, the overall texture content of samples with RH was lower than that of samples with SH.

## 4. Discussion

In the present investigation, the influence of heating rate, target temperature and holding time on the recrystallization behavior on high-purity Al-0.5%Cu plates was studied. This was established by continuous heating of cold-rolled samples within two different heating rate, target temperature from 200–300 °C and holding time from 2.5–80 min. Furthermore, it was evident that there seems to be a change in recrystallization mechanism under different annealing progress. In the following paragraphs, the microstructure evolution due to the differences in recrystallization behavior will be discussed.

**Figure 10:**
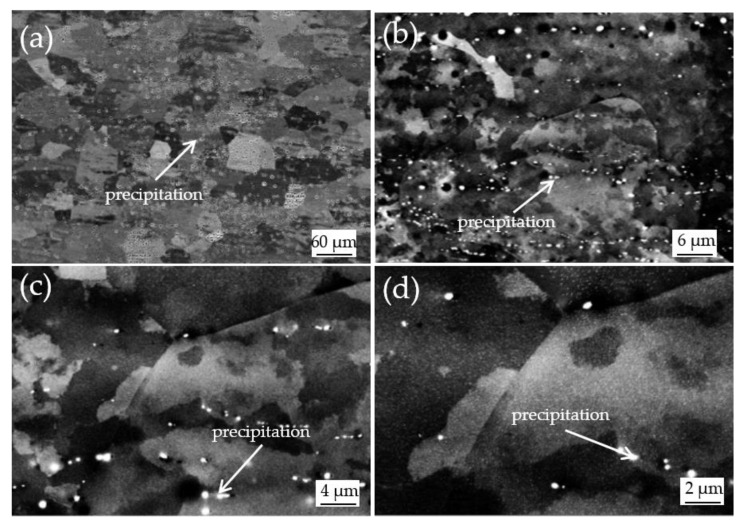
Precipitation distribution of Al-0.5%Cu alloy in SH annealing at 250 °C for 10 min in different multiples: (**a**) 500×; (**b**) 5000×; (**c**) 7000×; (**d**) 10,000×.

**Figure 11:**
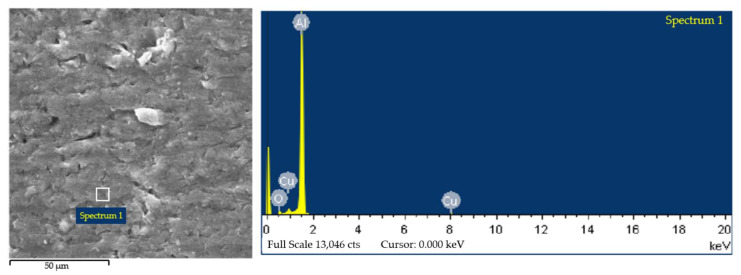
The EDS of precipitation particles of Al-0.5%Cu alloy in SH annealing at 250 °C for 10 min.

Experimental results observed in the present works revealed that the cold-rolled Al-0.5% Cu in SH possessed completely different grain structures and textures from the alloy in RH. In the case of SH, cold-rolled Al-0.5%Cu exhibited course grain structures, strong P and Cube-_ND_ recrystallization textures, while fine equiaxed grain structures, weaker Cube and P textures were observed in the case of RH. After annealing at 250 °C for 10 min in SH, there was a lot of second phase in the material (Figure 10). When the second phase was determined by EDS (Energy Dispersive Spectrometer), which can analyze the composition of tiny area of materials (Figure 11). It was found that there were Al, Cu and a small amount of oxygen elements in the second phase, which was Al_2_Cu phase precipitated in the progress of SH treatment according to preliminary analysis. The second phase particles have a great influence on the recrystallization texture and dislocation is easy to accumulate around the second phase particles during the deformation progress. When the size of the hard and brittle second phase particles in the matrix exceeds the critical size, the region of high dislocation density around the second phase particles will become the place where the recrystallization nucleation occurs preferentially. A large number of studies have shown that the second phase particles can promote the formation of P texture and Cube-_ND_ texture, which can reduce the content of Cube texture in the completely recrystallized texture.

Liu, W. C. et al. [28] used two annealing processes to study the influence of different heating rates on the recrystallization behavior of AA3015 aluminum alloy. They found that P texture and Cube-_ND_ texture were formed by SH treatment, while finer equiaxed grains and less Cube recrystallization textures were formed in RH. They interpreted this phenomenon as diffusion accompanied by precipitation during the annealing process and affected the grain size and texture type of the recrystallization structure. Wang, X. et al. [29] used two annealing processes with different heating rates to heat Al-Mg-Si-Cu and found that the grains produced by SH treatment were coarser and Cube-_ND_ texture was formed. They concluded that recrystallization texture and change of grain size were caused by the precipitation. Birol, Y. [30] found that at the beginning of annealing, the particles of the second phase would precipitate at the grain boundary, thus reducing the amount of nucleation in the recrystallization, and the precipitation of the second phase would lead to formation of P texture and Cube-_ND_ texture.

Humphreys, F. J. et al. [31] believed that the relationship between the second phase and recrystallization can be divided into three cases: the first is that the second phase precipitates before the recrystallization occurs; the second is that the second phase and the recrystallization occur simultaneously; the third is that the recrystallization occurs before the precipitation of the second phase. Therefore, different annealing processes will affect the relationship between recrystallization and precipitation of the second phase, resulting in the difference of recrystallization microstructure. Furthermore, they found that the recrystallized grains were elongated due to Zener pinning, which arose from precipitation on prior high angle grain boundaries. As heating rate increased, the amount of precipitation decreased and recrystallization occurred faster than precipitation, which created less interaction of the two processes. Therefore, the size of recrystallized grains was reduced, and recrystallized grains became equiaxed.

In this study, it was found that the heating rate had a more significant effect on grain size than target temperature and holding time. By comparing the recrystallized microstructure of the samples in the SH and RH treatment, it can be found that the RH can refine the recrystallized grain and make the grain distribution more uniform. According to the above SEM characterization, we found a large amount of precipitation, which can be preliminarily attributed to the generation of P texture and Cube-_ND_ texture and resulting in coarse recrystallized grains. However, the specific reasons need further study.

## 5. Conclusions

The effect of annealing process on microstructure, hardness, and texture of Al-0.5%Cu alloy was investigated. These following conclusions were drawn from this research:

(1) The recrystallization microstructure of the alloy was greatly influenced by heating rate. The RH samples were comprised of equiaxed grains with finer size, while the SH samples were composed of coarse grains with higher length-width ratio;
(2) Hardness of the alloy in process of recovery and recrystallization was affected by annealing processes. The RH samples were faster than SH samples at decreasing hardness and the time for completing recrystallization of RH samples was shorter;
(3) The recrystallization texture of the alloy was also significantly affected by applied annealing process. Higher temperatures and holding time promoted texture transformation and made textures more random. After completing recrystallization, the distribution of textures did not change with a change in holding time. Different heating rate affected the strength of texture components, samples in RH had lower texture strength and more random texture distribution.

## Figures and Tables

**Table 1:** Main texture components of FCC metals.

Texture Type	Miller Index	Euler Angle (φ_1_, Φ, φ_2_)		
Cube	{001} <100>	0	0	0
Cube-_RD_	{013} <100>	0	19	0
Cube-_ND_	{001} <310>	19	0	0
Goss	{011} <100>	0	45	0
P	{011} <122>	70	45	0
Brass	{011} <211>	35	45	0
Copper	{112} <111>	90	35	45

## Data Availability

The data presented in this study are available on request from the corresponding author.
